# Reproductive interference in live-bearing fish: the male guppy is a potential biological agent for eradicating invasive mosquitofish

**DOI:** 10.1038/s41598-019-41858-y

**Published:** 2019-04-01

**Authors:** K. Tsurui-Sato, S. Fujimoto, O. Deki, T. Suzuki, H. Tatsuta, K. Tsuji

**Affiliations:** 1 Center for Strategic Research Project, University of the Ryukyus, Sembaru, Nishihara, Okinawa, 903-0213 Japan; 2 Department of Agro-Environmental Sciences, Faculty of Agriculture, University of the Ryukyus, Sembaru, Nishihara, Okinawa, 903-0213 Japan; 3 The United Graduate School of Agricultural Sciences, Kagoshima University, Korimoto 1-21-24, Kagoshima, 890-8580 Japan

## Abstract

The eradication of invasive exotic species is desirable but often infeasible. Here, we show that male guppies are a potential biological agent for eradicating invasive mosquitofish through the mechanism of reproductive interference, which is defined as any sexual behavior erratically directed at a different species that damages female and/or male fitness. Together with decades of data on species distribution, our field surveys suggest that mosquitofish initially became established on Okinawa Island before being replaced by the more recently introduced guppies. More importantly, our laboratory experiments suggest that reproductive interference was one of the mechanisms underlying this species exclusion, and that in this case, the negative effects were asymmetric, i.e., they only impacted mosquitofish. Reproductive interference may offer a safer and more convenient method of biological control than the traditional sterile male release method because radiation is not necessary.

## Introduction

Biological invasions are a major threat to biodiversity and ecosystem functioning around the world. To control these invasions, environmentally friendly methods are desired. In one such method, sterile male release (i.e., the sterile insect technique^1,2^), males of the focal pest species are mass-reared, sterilized with radiation, and released over pre-defined areas. Because the wild females they mate with produce no offspring, this can lead to the extinction of the pest population^1^. The environmentally friendly nature of the sterile insect technique is contingent on the selectivity of sterile males in mating only with conspecific females. However, recent studies have reported that males can misidentify their reproductive partners; consequently, male reproductive behavior toward females of a different species is often observed in a broad range of taxonomic groups^3^. Such heterospecific sexual interactions can reduce the fitness of heterospecific females, and may provide new opportunities for biological pest control^4,5^.

Reproductive interference is defined as any kind of interspecific interaction during the process of mate acquisition that is caused by such as incomplete species recognition and/or signal jamming, and which adversely affects the fitness of at least one of the species involved^4,6^. In principle, reproductive interference is possible at any stage of mate acquisition, from signaling to copulation to fertilization. As with competition, reproductive interference can lead to the displacement of one species (sexual exclusion), spatial, temporal, or habitat segregation, changes in life history parameters, and reproductive character displacement^4^. Reproductive interference has been reported in various taxa such as plants^7^, insects^8^, frogs^9^, and geckos^10^. It often occurs between closely related species^4^, because they may share similar sexual signals owing to common ancestry^11^. However, reproductive interference also occurs between distantly related taxa^12^, in particular between allopatric species^13^. Its presumable mechanism is the prolonged absence of the selective pressures that drive signal divergence^14^. Hence, reproductive interference between secondarily contacted allopatric species, including invasive and native species, has received much attention^4^.

The mosquitofish *Gambusia affinis* (Cyprinodontiformes:Poeciliidae) was introduced worldwide from its native range in eastern North America starting in the early twentieth century to control mosquito populations and the human diseases they carry^15^. In contrast to the mosquitofish’s purported ability to control mosquito populations, their negative impacts on many native species are supported by abundant evidence^16^. The guppy *Poecilia reticulata* has also been introduced all over the world to control mosquitoes in tropical countries^17^. The guppy is a popular aquarium fish, and its natural range appears to be in Trinidad, Venezuela, Guyana, Surinam, and probably Tobago^17–19^. Guppies have successfully become established in many countries such as those in the Americas, Europe, Asia, Australia, and Africa^20^ via the accidental or deliberate release of artificially bred lineages^21^.

**Figure 1:**
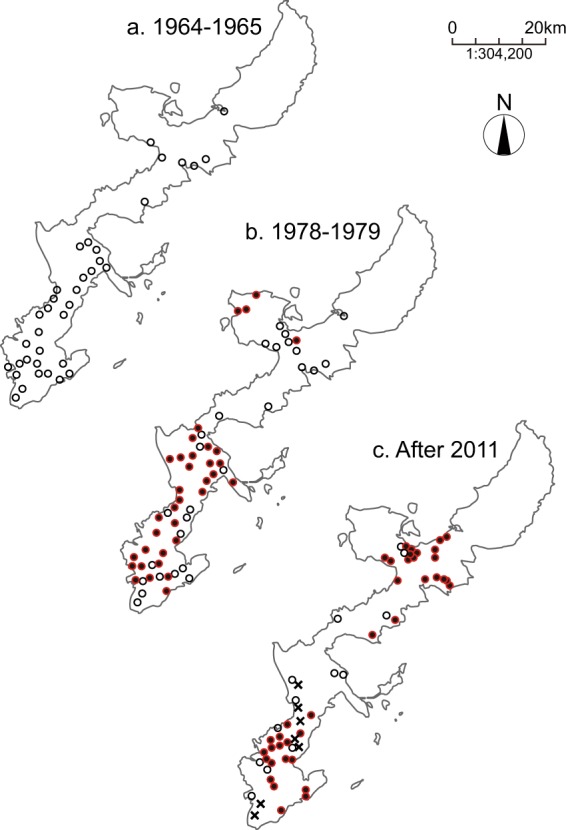
The distribution of mosquitofish and guppies on Okinawa Island during (**a**) 1964–1965 (data from Kochi, 2003^23^), (**b**) 1978–1979 (data from Kochi, 2003^23^), and (**c**) after 2011 (data from Ohsumi *et al*., 2014^54^ and present study). Open circles indicate the presence of mosquitofish, filled circles indicate the presence of guppies and cross marks indicate where both species were found.

Mosquitofish were introduced into Okinawa Island (the main island of the Ryukyus), Japan (26°7′N, 127°42′E) around the beginning of the twentieth century through Hawaii and Taiwan^22^. The spread of mosquitofish across Okinawa Island up to the 1960s led to the local extinction of indigenous fish such as the Japanese medaka *Oryzias latipes*^23^ (Fig. 1a). However, after the introduction of guppies in the 1960s, mosquitofish populations reportedly started to be replaced by guppies in the late 1970s^23^ (Fig. 1a,b). At present, both mosquitofish and guppies are found on Okinawa Island^24^. These lineages of mosquitofish and wild guppy are likely to have contacted each other for the first time on Okinawa Island, because their original ranges do not overlap.

Male guppies attempt to mate with other poeciliid females, including *Poecilia picta*^25^, as well as viviparous goodeids such as *Skiffia bilineata* (Cyprinodontiformes:Goodeidae)^26^. We suspected that reproductive interference may occur between guppies and mosquitofish because females of the two fish appear morphologically similar despite their phylogenetic distance (they belong to different genera), and because the two species share visual mate recognition and choice systems (for the guppy, see^17^; for the mosquitofish, see^27^). Moreover, both species are livebearers and males have gonopodiums for internal fertilization. In fact our preliminary observations have confirmed that males of both species attempt coercive mating with heterospecific as well as conspecific females (See Movie S1 for footage of coercive mating by a guppy male on a mosquitofish female, and Movie S2 for footage of a mosquitofish male on a guppy female).

In the present study, we hypothesized that reproductive interference underlies the replacement of mosquitofish by guppies on Okinawa Island. We tested this hypothesis in one observational field study and two laboratory experiments. First, we investigated the distribution of the two species in the field on Okinawa Island, and conducted a statistical analysis to evaluate any exclusion effects. Second, we examined reproductive interference between the two species in the laboratory by focusing on female fecundity. Here, we predicted that the negative effects of reproductive interference would be unidirectional, namely, they would only impact mosquitofish. Last, we also conducted laboratory tests to assess whether this reproductive interference by guppies reduces the population growth rate of mosquitofish. Based on these data, we propose a possible new control method of invasive mosquitofish based on reproductive interference by guppies.

## Results

### The distributions of *G*. *affinis* and *P*. *reticulata* on Okinawa Island

**Figure 2:**
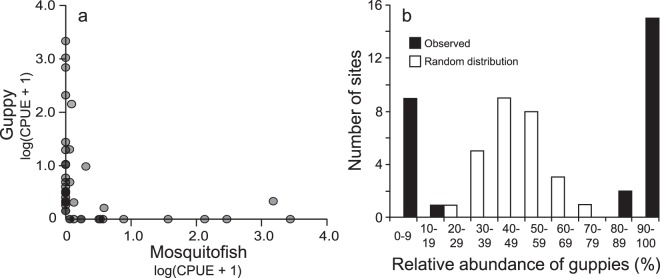
Patterns of mosquitofish and guppy coexistence/exclusion. (**a**) Each point indicates log (CPUE + 1) for mosquitofish and guppies at a survey site (*n* = 41), where CPUE represents catch per unit effort. (**b**) Frequency distribution of the relative abundance of guppies at the survey sites (*n* = 27). White bars show the null frequency distribution assuming the two species are randomly distributed, and black bars show the observed one.

To assess any exclusion effects between mosquitofish and guppies, we investigated their distributions on Okinawa Island in 2015 (Fig. 1c). Spearman’s rank correlation test revealed a significant negative relationship between mosquitofish and guppy catch per unit effort (CPUE: a proxy for density) (Spearman’s rank correlation coefficient, ρ = −0.647, a lower and an upper 95% CI of ρ, −0.783 and −0.448, S = 18913, *df* = 39, *p* < 0.001, Fig. 2a).

The observed frequency distribution of the relative abundance of guppies (number of guppies divided by the total number of guppies and mosquitofish) showed a concave distribution. At most sites, the proportion of guppies was less than 10% or over 90%. In contrast, the null frequency distribution assuming a random distribution of the two fish species showed a convex distribution with a peak at around 50%. Fisher’s exact test of independence confirmed that the distribution of the two fish species was nonrandom, suggesting the existence of exclusion effects (*df* = 9, *p* < 0.001, Fig. 2b).

### Experiment 1: Individual fitness costs of reproductive interference

**Figure 3:**
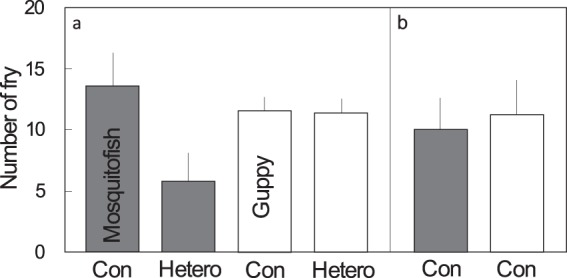
The results of Laboratory Experiment 1 (individual fitness costs of reproductive interference). (**a**) Number of fry produced under the main treatments, *conspecific* (con) and *heterospecific* (hetero), for groups of three fish, for each species (*n* = 8, 9, 10, and 7, respectively). (**b**) Number of fry produced under the additional treatment, *conspecific*, for groups of two fish, for each species (*n* = 7 and 7, respectively). Grey bars indicate mosquitofish and white bars indicate guppies. Error bars indicate mean ± SE. For a summary of the treatments see Table 1.

Whether heterospecific reproductive interactions between mosquitofish and guppies reduce female fitness was examined at the individual level (for a summary of treatments see Table 1a). Mosquitofish females had lower fecundity in the presence of a heterospecific male, whereas, remarkably, guppy females did not (Fig. 3a). The above observation was supported by a generalized linear model analysis of female fecundity, in which a statistically significant interaction effect of the female species and the presence or absence of a heterospecific male was detected (estimated fixed effects: female species [mosquitofish to guppy]: e^Coef^ = 1.766, e^SE^ = 0.133, *z* = 4.264, *df* = 1, *p* < 0.001; male treatment [heterospecific to conspecific {i.e., presence or absence of a heterospecific male}]: e^Coef^ = 1.080, e^SE^ = 0.145, *z* = 0.527, *df* = 1, *p* = 0.598, interaction [mosquitofish to guppy × heterospecific to conspecific] e^Coef^ = 0.424, e^SE^ = 0.223, *z* = −3.851, *df* = 1, *p* < 0.001; Fig. 3a). This was not due to the putative effect of the absolute number of conspecific males on female reproductive success, because the number of conspecific males did not significantly influence female fecundity in either female species (estimated fixed effects: number of conspecific males [trio to pair] for mosquitofish: e^Coef^ = 1.347, e^SE^ = 0.153, *z* = 1.946, *df* = 1, *p* = 0.052; number of conspecific males [trio to pair] for guppies: e^Coef^ = 1.013, e^SE^ = 0.146, *z* = 0.086, *p* = 0.931; Fig. 3a,b). In sum, these results clearly show unidirectional reproductive interference on mosquitofish by guppies.

**Table 1:** Summary of treatments for experiments 1 and 2.

Female species	Male treatment	Mosquitofish female	Guppy female	Mosquitofish male	Guppy male	Sample size
**(a) Laboratory Experiment 1: Individual fitness costs of reproductive interference**						
Main treatment (trio)^c^						
Mosquitofish	*Conspecific* ^a^	1	0	2	0	8
	*Heterospecific* ^b^	1	0	1	1	9
Guppy	*Conspecific*	0	1	0	2	10
	*Heterospecific*	0	1	1	1	7
Additional treatment (pair)^c^						
Mosquitofish	*Conspecific*	1	0	1	0	7
Guppy	*Conspecific*	0	1	0	1	7
**(b) Laboratory Experiment 2: Effects of reproductive interference at the group level**						
Main treatment (8 males)^c^						
Mosquitofish	*Conspecific*	6	0	8	0	6
	*Heterospecific*	6	0	4	4	6
Guppy	*Conspecific*	0	6	0	8	6
	*Heterospecific*	0	6	4	4	6
Additional treatment (4 males)^c^						
Mosquitofish	*Conspecific*	6	0	4	0	6
Guppy	*Conspecific*	0	6	0	4	6

^a^Heterospecific male absent.

^b^Heterospecific male present.

^c^The main treatments control for total fish density, whereas additional treatments set the conspecific male density equal to that in the heterospecific main treatment.

### Experiment 2: Effects of reproductive interference at the group level

**Figure 4:**
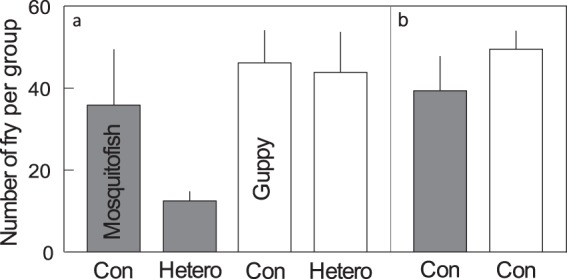
The results of Laboratory Experiment 2 (reproductive interference at the group level). The total number of fry produced per aquarium over five weeks under (**a**) the main treatments, *conspecific* (con) and *heterospecific* (hetero), for groups of 14 fish, for each species; and (**b**) an additional treatment, *conspecific*, for groups of 10 fish, for each species (*n* = 6 for all treatments). Grey bars indicate mosquitofish and white bars indicate guppies. Error bars indicate mean ± SE. For a summary of the treatments see Table 1.

Experiment 2, which repeated the procedures from Experiment 1 at a higher fish density, showed that unidirectional reproductive interference was also present at the group level (see Table 1b for a summary of treatments). Whereas mosquitofish females experienced lower group reproductive rates (the total number of fry produced by a group of females over five weeks) in the presence of heterospecific males, guppy females did not (Fig. 4a). This observation was supported by our generalized linear model, which showed a significant interaction effect between female species and the presence or absence of heterospecific males (estimated fixed effects: female species [mosquitofish to guppy]: e^Coef^ = 0.741, e^SE^ = 1.095, *z* = −3.302, *df* = 1, *p* = 0.001; male treatment [heterospecific to conspecific {presence or absence of a heterospecific male}]: e^Coef^ = 0.875, e^SE^ = 1.090, *z* = −1.556, *df* = 1, *p* = 0.120; interaction [mosquitofish to guppy × heterospecific to conspecific]: e^Coef^ = 0.431, e^SE^ = 1.173, *z* = −5.282, *df* = 1, *p* < 0.001; Fig. 4a). This was not due to the putative effect of fish density on female reproductive success (estimated fixed effects: number of conspecific males [eight to four] for mosquitofish: e^Coef^ = 0.946, e^SE^ = 1.099, *z* = −0.591, *df* = 1, *p* = 0.554; number of conspecific males [eight to four] for guppies: e^Coef^ = 0.991, e^SE^ = 1.087, *z* = −0.109, *p* = 0.931; Fig. 4a,b). Moreover, female body weight gain in either species was affected by neither fish density (estimated fixed effects: number of conspecific males [eight to four] for mosquitofish: Coef = 0.020, SE = 0.061, *t* = 0.335, *df* = 1, *p* = 0.732; number of conspecific males [eight to four] for guppies: Coef = 0.049, SE = 0.064, *t* = 0.772, *p* = 0.398) nor the presence or absence of heterospecific males (estimated fixed effects: female species [mosquitofish to guppy]: Coef = 0.137, SE = 0.059, *t = *2.305, *df* = 1, *p* = 1.000; male treatment [heterospecific to conspecific {presence or absence of a heterospecific male}]: Coef = 0.036, SE = 0.059, *t* = 0.601, *df* = 1, *p* = 1.000; interaction [mosquitofish to guppy × heterospecific to conspecific]: Coef = −0.118, SE = 0.084, *z = *−1.410, *df* = 1, *p* = 0.154) during the experimental period.

Taken together, these results demonstrate that male guppies unidirectionally reduced the reproductive rate of mosquitofish groups without affecting the body weight of female mosquitofish.

## Discussion

Our field surveys show that mosquitofish and guppies exclude each other at the microhabitat level (area within a 50-m radius). Even where the two species coexist, the relative species abundance was skewed toward one or the other species (Fig. 2). This information, together with decades of data on species distributions (Fig. 1), strongly suggests the following history: mosquitofish were introduced to Okinawa Island first, and became widespread on the island, but after the introduction of guppies, they were eventually replaced. More importantly, our laboratory experiments suggest that reproductive interference is one of the mechanisms that underlie this species exclusion. Specifically, we detected asymmetric negative effects, i.e., the reproductive interference existed from guppies to mosquitofish but not from mosquitofish to guppies (Figs 3 and 4). As Smith (2007)^28^ reported that male population density does not influence the fitness of mosquitofish females, the difference in the number of offspring between treatments in Laboratory Experiment 2 was presumably caused by the presence or absence of guppy males rather than by differences in fish density. The results of Laboratory Experiment 1 also confirmed that the number of conspecific males did not influence female fecundity in mosquitofish or guppies (Fig. 3a,b, Table S2).

The reduction of female fitness by reproductive interference could potentially be caused by any interspecific interactions during mate acquisition, such as signal jamming during mate attraction, heterospecific rivalry, misdirected courtship, heterospecific mating attempts, erroneous female choice, heterospecific mating, or hybridization^4^. We have behavioral evidence that misdirected courtship and heterospecific mating attempts by males on heterospecific females occur between the two species in both directions (Movies S1 and S2). Interestingly, Experiment 2 showed that adding male guppies to mosquitofish groups did not affect the body weight of female mosquitofish (Table S4). This suggests that the adverse effects of reproductive interference by guppies on mosquitofish were caused by hindering normal fertilization rather than by the cost of sexual harassment, such as in decreased feeding time^29^. Admittedly, further studies are necessary to better understand the mechanism of reproductive interference, especially to determine why this negative effect was unidirectional, i.e., only affecting mosquitofish but not guppies. In addition to more behavioral observation, detailed investigations of the effects of anatomy and physiology on insemination and fertilization will be key.

Other environmental factors such as water velocity, water temperature, turbidity, degree of water system isolation, and concentration of dissolved chemicals and oxygen may also influence guppy and mosquitofish abundance in the wild. In addition, anthropogenic impacts such as water pollution may also be a factor^22^, because the replacement of mosquitofish by guppies in Okinawa Island appears to be associated with urbanization. The effects of these factors on the abundance of mosquitofish and guppies should be explored in the future.

Furthermore, it is possible for other mechanisms to drive competitive displacement. Despite the increasing attention paid to reproductive interference, resource competition remains a central hypothesis for understanding competitive displacement^30^. In addition, differential rates of reproduction and intra-guild predation in carnivorous species are also important drivers of competitive displacement^30^. Several different mechanisms can affect competitive displacement in a synergistic manner (for reproductive interference and resource competition, see^31^; for reproductive interference and intra-guild-predation, see^32,33^).

### Exclusion by guppies: Risks and possible applications

Mosquitofish and guppies are both regarded as effective biological agents for controlling mosquito populations^16,17^. As a result, the two species have been introduced in, and have successfully colonized, broad areas of the world. As far as we know, this is the first report of mosquitofish exclusion by guppies within or outside their natural ranges. It is of great interest to test whether such exclusion also occurs in other geographical regions outside Okinawa Island, especially within the native mosquitofish range. Our findings also highlight the risk of invasive guppies replacing other poeciliid fish including endangered native species.

On the other hand, this reproductive interference could be useful for pest control efforts. It could be the basis for a biological control method with high species-specificity similar to the sterile male release technique^5^. Although the sterile male release technique has been successful in eradicating a number of pest populations such as the screwworm (*Cochliomyia hominivorax*) and melon fly (*Bactrocera cucurbitae*), it usually comes at a prohibitively high cost, partly due to the use of radiation facilities^34,35^. Reproductive interference is a potential alternative to the sterile male release technique across a broad range of taxa because reproductive interference is now known to be widespread from angiosperms to vertebrates^36^.

There is, however, concern that biological agents may negatively impact non-target species through processes such as predation and competition including reproductive interference^5,16,37^. Any proposal to control mosquito populations by releasing guppies into the wild entails some risk that the guppies may become invasive and damage local ecosystems and biodiversity^38^. For example, within 25 years, experimentally-introduced guppies caused ecological and evolutionary changes in the population densities and life history traits of killifish, *Rivulus hartii*^39^. Although guppies are a widespread invasive fish, the likelihood of introduced guppies becoming invasive and the mechanisms of ecosystem impact are little understood (but see^39–42^). It is crucial to accumulate behavioral, evolutionary, and ecological knowledge on invasive guppies all over the world to formulate appropriate countermeasures against the invasive spread of both intentionally introduced and accidentally released guppies.

In using guppies as biological agents, it is essential to prevent their establishment in the local habitat in order to limit environmental impacts. One way to do so could be to only release male guppies into the environment. In temperate regions, both sexes could even be released with limited environmental impact, because the guppies will die off during the winter (the annual water temperature range in the guppy’s native range is 18–28 °C^20^). Even under these situations, however, potential ecological risks should be carefully assessed before any releases are conducted. Risks that should be considered include predation on juvenile non-target fish, genetic introgression, reproductive interference by male guppies on other native poeciliid fish, and the likelihood of establishment. A further practical obstacle for this method is the mass rearing of only male guppies. We believe, however, that these problems can be overcome, as the genetic and ecological understanding required for the quality control of genetic traits and a planned release strategy in guppies^43–47^ have been extensively studied for more than 80 years^48^. Our findings suggest that guppies, which have heretofore served as model organisms for sexual selection and as popular ornamental pets, can also serve as biological agents for controlling invasive mosquitofish.

## Methods

### Field surveys

We chose a survey site in each of 28 independent drainage systems and 12 ponds. For long rivers more than 2 km, of which there were eight, we chose additional survey sites. Thus, we chose 50 sites in total. The survey area at each site was less than 50 m in radius. Adult mosquitofish and guppies were captured by hand net (mesh size, 2 mm; capture area, 770 cm^2^; shaft, 60 cm) by one of the authors (T. Suzuki). The capture period ended once approximately 60 fish had been captured, and the duration of the capture period was recorded. We then calculated the catch per unit effort (CPUE) for each species at each site by dividing the number of captured fish by the duration of the capture period. For low-density populations, the capture period was limited to 30 minutes. If no fish were captured within 30 minutes, we designated the CPUE of both species as zero and excluded the data from the analyses.

### Laboratory experiments

#### General methods

We collected mosquitofish from an isolated artificial pond (26.25°N, 127.76°E) on Okinawa Island, Japan. Guppies were collected from Makiminato River (26.25°N, 127.74°E), also on Okinawa Island, and a nearby ditch (26.25°N, 127.76°E). The collected fish were separated by species and allowed to acclimate in the laboratory (26 ± 1 °C and natural day length) in stock aquariums (30 × 40 × 60 cm). Water in the aquariums was continuously circulated and filtered. The fish were fed daily with dry foods (Hikari Tropical Fancy Guppy, Kyorin Co., Ltd., Himeji, Japan) (approximately 5% of fish body weight) and frozen Chironomidae larvae (UV Akamushi, Kyorin Co., Ltd., Himeji, Japan). Because adults are known to cannibalize juveniles, we placed a floating refuge made of artificial turf mats (Midushima Industry Co. Ltd., Osaka, Japan) into which fry could infiltrate but adults could not (overall size, 30 × 60 × 2 cm; mesh size approximately 3 mm) into each aquarium (Fig. S1). Each experimental fish was sexed based on anal fin shape^49^. The maximum density of fish in experimental aquaria (78 fish/m^2^) was within naturally observed limits on Okinawa Island for all experiments. We followed the experimental method of Smith and Sargent (2006)^50^ with some modifications for Laboratory Experiment 2.

#### Laboratory Experiment 1: Individual fitness costs of reproductive interference

We used virgin matured females that were F1 born in the laboratory and reared individually until the experiments began. The males were sexually mature individuals collected in the wild that were allowed to acclimate to laboratory conditions for about two weeks prior to the following experiments. Each guppy or mosquitofish female was reared with two conspecific males or two mixed males (a guppy and mosquitofish male) in identically sized glass aquaria (21 × 25 × 29 cm). In order to test the effect of the number of conspecific males, we also prepared two additional treatments: a guppy or mosquitofish female with one conspecific male, respectively. The treatments are summarized in Table 1a. Each treatment was replicated 10 times and the experiment ran for 12 weeks between June and September 2017. The number of fry at first birth for each female was recorded. When the females did not give birth within 12 weeks, the trial was terminated and the number of fry was recorded as zero. Females that died during experiments were excluded from the analyses. When males died during experiments, replacement males were added to the aquaria. At the end of the experiment, males and females were weighed, euthanized according to our IACUC protocol with tricaine methane sulfonate (MS-222), and fixed in 10% formalin.

#### Laboratory Experiment 2: Effects of reproductive interference at the group level

We used sexually mature individuals collected in the wild. Fish were allowed to acclimate to laboratory conditions for 6 weeks prior to the following experiments. Female mosquitofish and guppies were individually discriminated by Visible Implant Elastomer Tags (Northwest Marine Technology, Inc., Shaw Island, WA, USA) and measured (wet weight). We did not discriminate individual males. Fish were grouped into glass aquaria (30 × 40 × 60 cm) according to six different treatments. Each group of six guppy or mosquitofish females was reared with eight conspecific males or eight mixed males (four guppies and four mosquitofish). In order to test the effect of the number of conspecific males, we also prepared two additional treatments: six guppy or mosquitofish females with four conspecific males. The treatments are summarized in Table 1b. Each treatment was replicated 6 times, and the experiment ran for 11 weeks from March to June 2018. Newly born fry were counted and removed every day with a hand net. When fish died during the experiment, replacement fish were added within 24 hours to maintain a constant fish density. Replacement females were individually discriminated and measured (wet weight), but replacement males were not. Only five mosquitofish females and three guppy females died during the 11-week experiment. There was no aquarium in which two or more females died.

We used fry production data from the last five weeks of the experiment, because fry produced during the first six weeks may have been conceived in the wild. Mosquitofish females produce clutches continuously throughout the breeding season at approximately 30-day intervals for each individual^51,52^, allowing all females the opportunity to give birth at least once during the last five weeks. The total number of fry collected in each aquarium was calculated as the number of fry born per aquarium minus any mortality that occurred before collection. At the end of the experiment, females were weighed, euthanized according to our IACUC protocol with tricaine methane sulfonate (MS-222), and fixed in 10% formalin. When a fish died during the 11-week period, we measured its body weight within 24 hours. We also measured the body weights of replacement fish on the day of introduction and on the final day of the experiment. We defined female body weight as the mean of an individual’s initial and final wet weights. We also defined female body weight gain as an individual’s final wet weight minus its initial wet weight. The per-aquarium averages of female body weight and female body weight gain were weighted by the residence time in days of each fish in the aquarium (females that died and were replaced had a shorter residence time) and used in the analyses for Experiment 2.

#### Statistical analyses

We tested the independence of the distribution of mosquitofish and that of guppies in two ways. First, the CPUEs of the two species at 41 sites were analyzed using the Spearman’s rank correlation method. A two-sided test was conducted to examine the significance of the association. The confidence interval of a Spearman’s rank correlation coefficient was computed by bootstrapping (1000 replicates for bootstrapping). If the two species were independently distributed, no correlation would be expected. The sites with no captured individuals of the focal species (nine sites) were excluded from the first analysis. Second, we used the two-sided Fisher’s exact test of independence, in which we assumed the null hypothesis that individuals were randomly distributed regardless of species. It was also assumed that all sites had the same relative species abundance of guppies (i.e., the density of guppies divided by the total density of mosquitofish and guppies). The expected frequency distribution of the relative species abundance of guppies under the null hypothesis was calculated at 10% intervals using a binomial distribution (where the sample size, i.e., 20 fish, is equal at all sites), and compared with measured site-frequency data. Sites with more than 20 captured individuals of the focal species (27 of 50 sites) were used in the second analysis. Twenty captured individuals of the focal species were randomly resampled from original data for each sample.

For the main treatments in Laboratory Experiment 1, we constructed a generalized linear model (GLM) with a Poisson error structure and a log link. The model included the number of fry (female fecundity) as a response variable, female species, treatment (presence or absence of a heterospecific male), and their interactions as fixed effects, and female body weight as an offset. In addition, to investigate the effect of conspecific male density on female fecundity, we constructed GLMs with a Poisson error structure and a log link for each species without using data from heterospecific treatments. The models for each species included the number of fry (female fecundity) as a response variable, treatment (one or two conspecific males) as a fixed effect, and female body weight as an offset. The significance of the fixed effects was tested using the one-sided Wald test.

For the main treatments in Laboratory Experiment 2, we constructed a GLM with a Poisson error structure and log link. The model included the total number of fry born in the last five weeks in each aquarium (group birth rate) as a response variable, female species, treatment (presence or absence of a heterospecific male), and their interactions as fixed effects, and mean female body weight per aquarium as an offset. In addition, to investigate the effect of conspecific male density on female fecundity, GLMs with a Poisson error structure and log link were constructed for each species without using the data from heterospecific treatments. Models for each species included the total number of fry born during the last five weeks in each aquarium (group birth rate) as a response variable, treatment (four or eight conspecific males) as a fixed effect, and mean female body weight in each aquarium as an offset. The significance of the fixed effects was tested using the one-sided Wald test.

The effect of the treatments on female body weight gain in Laboratory Experiment 2 was also investigated using generalized linear mixed models with a Gaussian error structure and identity link. For the main treatments, the model included individual female body weight gain as a response variable, female species, treatment (presence or absence of a heterospecific male), and their interactions as fixed effects, and aquarium ID as a random effect. In addition, to investigate the effect of conspecific male density on female body weight gain, generalized linear mixed models with a Gaussian error structure and identity link were constructed for each species without using the data from heterospecific treatments. Models for each species included individual female body weight gain as a response variable, treatment (four or eight conspecific males) as a fixed effect, and aquarium ID as a random effect. To examine the significance of each fixed effect, we performed likelihood-ratio tests (one-sided) to compare full and reduced models.

All analyses described above were performed using R version 3.4.2^53^.

#### Ethical approval

All applicable institutional and/or national guidelines for the care and use of animals were followed. Permission for transporting and rearing *Gambusia affinis* was obtained from the Ministry of the Environment, Japan (Permit number: 15000146), and permission for conducting animal experiments with *Gambusia affinis* and *Poecilia reticulata* was obtained from the University of the Ryukyus (Permit number: A2017108).

## Supplementary information

Movie S1

Movie S2

Supplementary figure

Dataset 1

Dataset 2

Dataset 3

Dataset 4

Dataset 5

## Data Availability

All data generated or analyzed during this study are included in this published article (and its Supplementary Dataset Files).
