# Supplementary material for: Reproductive interference in live-bearing fish: the male guppy is a potential biological agent for eradicating invasive mosquitofish
Source: Sci Rep. 2019 Apr 1;9:5439. doi: 10.1038/s41598-019-41858-y (PMC6443680; doi:10.1038/s41598-019-41858-y)
Supplement: Supplementary file 3 — Supplementary figure [file 41598_2019_41858_MOESM3_ESM.pdf]

Supplementary figure

# Reproductive interference in live-bearing fish: the male guppy is a potential biological agent for eradicating invasive mosquitofish

**Author names and affiliations:**

K. Tsurui-Sato,[1*†] S. Fujimoto,[1†] O. Deki,[2†] T. Suzuki,[2] H. Tatsuta,[2,3] K. Tsuji[2, 3]

[1]Center for Strategic Research Project, University of the Ryukyus, Senbaru, Nishihara, Okinawa 903-0213, Japan.
[2]Department of Agro-Environmental Sciences, Faculty of Agriculture, University of the Ryukyus, Senbaru, Nishihara, Okinawa 903-0213, Japan.
[3]The United Graduate School of Agricultural Sciences, Kagoshima University, Korimoto 1-21-24, Kagoshima 890-8580, Japan.

*Corresponding author: Kaori Tsurui-Sato; Tel: +81-98-886-4191; Fax: +81-098-887-7188; Email: tsuruikaori@gmail.com

† These authors contributed equally to this work.

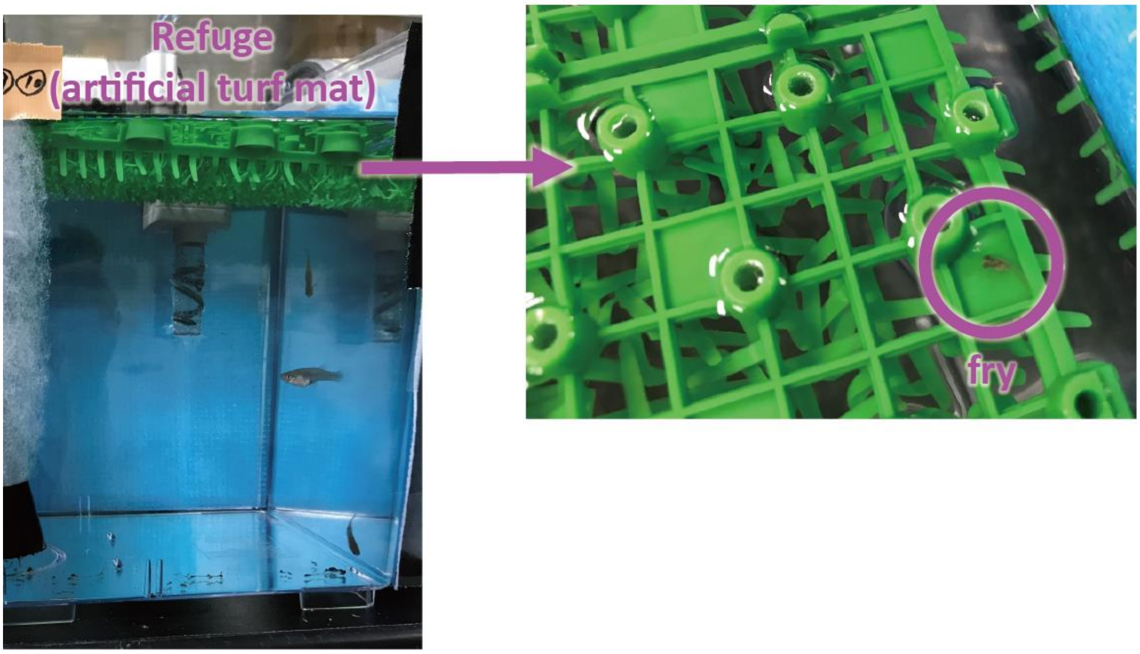


Fig. S1.
Photographs of the floating refuge for fry. Fry are able to infiltrate the turf mat, but adults are not.
